# Tumor exosomal miR-221-3p induces glycolysis through the LIFR/GLUT1 pathway to destroy the cerebral vascular endothelial cell barrier and promote breast cancer brain metastasis

**DOI:** 10.1186/s12967-025-07372-8

**Published:** 2025-11-21

**Authors:** Kaitao Zhu, Hongru Yao, Jilong Hei, Shiwei Li, Tongxin Ye, WenG Jiang, Shuwen Wang, Zhuojun Luo, Tracey Martin, Jie Zhou, Shanyi Zhang

**Affiliations:** 1 Department of Neurosurgery, Sun Yat-Sen Memorial Hospital, Sun Yat-Sen University, Guangzhou, China; 2 Guangdong Provincial Key Laboratory of Malignant Tumor Epigenetics and Gene Regulation, Guangdong-Hong Kong Joint Laboratory for RNA Medicine, Sun Yat-Sen Memorial Hospital, Sun Yat-Sen University, Guangzhou, China; 3 Department of Neurosurgery, HeYou International Health System, Foshan, China; 4 Cardiff China Medical Research Collaborative (CCMRC), (Cardiff University - Peking University Cancer Institute and Cardiff University - Capital Medical University Joint Centre Biomedical Research), Cardiff University, Cardiff, UK; 5 Department of Neurosurgery, The People’s Hospital of Fengqing, Lincang, China; 6 Department of Breast Oncology Surgery, Guangzhou Institute of Cancer Research, the Affiliated Cancer Hospital, Guangzhou Medical University, Guangzhou, 510095 China

**Keywords:** Exosome, miR-221-3p, BCBM, Glycolysis, LIFR, GLUT1, ZO-1, Occludin

## Abstract

**Background:**

Tumor-derived exosomal microRNAs mediate intercellular communication between malignant cells and distant organs and play a pivotal role in metastatic dissemination. Breast cancer brain metastasis (BCBM) poses a significant clinical challenge, with endothelial barrier dysfunction representing a critical yet poorly understood step in metastatic progression.

**Methods:**

A physiologically relevant in vitro blood‒brain barrier (BBB) model was established to evaluate exosomal functions. Mechanistic investigations included qPCR and western blotting for miR-221-3p, along with protein expression profiling, immunofluorescence-based tight junction protein visualization, apoptosis detection via annexin V/PI staining, EdU assays for proliferation quantification, and transendothelial migration assessments. To validate the underlying mechanism, ALIX/HRS were silenced to inhibit exosome secretion, and miR-221-3p antagonists were applied. Clinical relevance was assessed by analyzing plasma miR-221-3p levels in breast cancer (BC) patients.

**Results:**

Highly invasive breast cancer-derived exosomal miR-221-3p induced glycolysis and lactic acid accumulation in brain microvascular endothelial cells by targeting the leukemia inhibitory factor receptor (LIFR), leading to endothelial barrier destruction and reduced tight junction protein expression. This significantly enhanced endothelial barrier permeability and tumor cell transendothelial migration capacity. Silencing ALIX/HRS or antagonizing miR-221-3p markedly reversed these effects.

**Conclusions:**

Our findings indicate that BC can target LIFR in hCMEC/D3 cells via exosomal miR-221-3p, thereby promoting glycolysis and inhibiting the expression of tight junction proteins, which facilitates tumor metastasis.

**Graphical Abstract:**

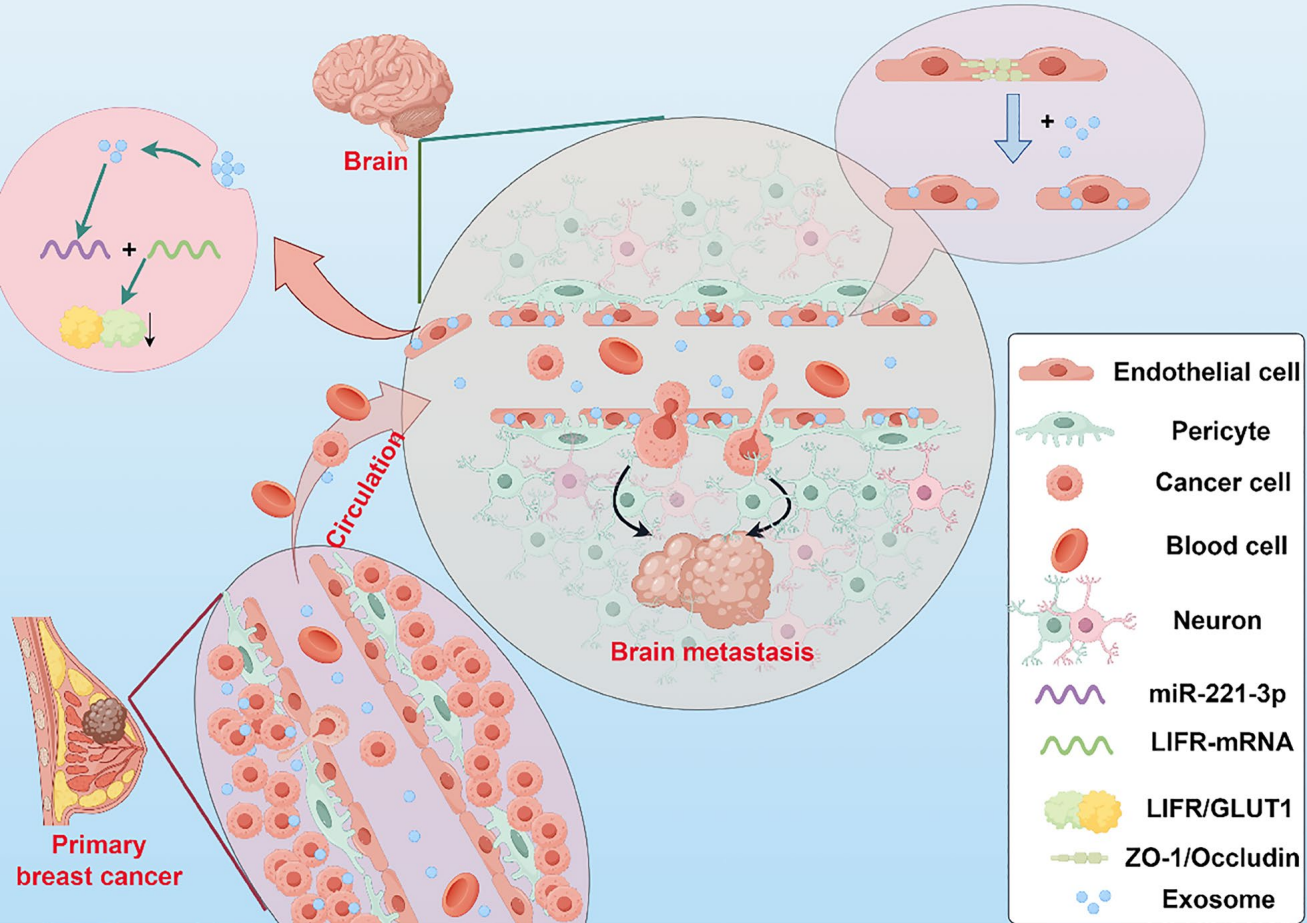

**Supplementary Information:**

The online version contains supplementary material available at 10.1186/s12967-025-07372-8.

## Background

Tumors metastasis is a prevalent cause of mortality among individuals with breast cancer (BC), and brain metastasis is linked to the poorest prognosis, yielding a median survival time of only 4 to 6 months [1, 2]. Despite significant advancements in BC treatment, most therapeutic agents fail to cross the blood–brain barrier (BBB) [3, 4], contributing to a persistently high incidence of brain metastasis in BC patients. This incidence is particularly pronounced in those with triple-negative breast cancer (TNBC), ranging from 30% to 50% [5, 6]. Currently, effective treatment options for breast cancer brain metastasis (BCBM) are lacking [7]. Consequently, research into the molecular mechanisms underlying BC, particularly in relation to TNBC brain metastasis, is urgently needed to increase the survival rates of affected patients.

Recent investigations have revealed that exosomes generated by malignant cells play an essential role in this mechanism. Tumor-derived exosomes can transport various bioactive molecules, including nucleic acids, lipids and proteins, to distant cells, thereby regulating their functions [8–12].

MicroRNAs (miRNAs) represent a category of tiny noncoding RNAs that control gene expression at the transcriptional stage and modulate various cellular activities [13–15]. The dysregulation of miRNA functions is linked to numerous pathological processes, particularly cancer development and progression [16, 17]. An increasing number of studies have demonstrated that miRNAs can be actively released and transported to recipient cells, where they influence cellular activities by inhibiting the translation of target genes. Moreover, miRNAs found in circulation have shown promise as prospective indicators for detecting and predicting outcomes in cancer patients [18–20].

The enhanced glycolytic capacity of tumor cells has emerged as a pivotal factor in tumorigenesis and tumor progression [21, 22]. Recent studies have also revealed that tumors can secrete bioactive factors, such as miRNAs, which remotely modulate the glycolytic capacity of cellular components within the metastatic microenvironment, thereby facilitating tumor metastasis [23–25]. Brain microvascular endothelial cells represent a crucial cell type within the BBB, and alterations in their glycolytic capacity can directly or indirectly influence the functionality of the BBB, subsequently facilitating the occurrence of tumor brain metastasis [26, 27]. However, research investigating the regulation of glycolytic metabolism in brain microvascular endothelial cells by BC remains limited. Therefore, conducting comprehensive studies on the interplay between BC and glycolysis in brain microvascular endothelial cells is essential for enhancing our knowledge of mechanisms underlying BCBM and developing novel treatment strategies.

## Methods

### Cell lines

The following cell lines were obtained from the American Type Culture Collection (ATCC): the breast cancer cell lines MDA-MB-231 and MCF-7; the non-tumorigenic human breast epithelial cell line MCF-10A, used as a normal control; as well as the human glioblastoma cell line U87 and the immortalized brain microvascular endothelial cells (hCMEC/D3). The cells were maintained at 37 °C with 5% carbon dioxide in an environment containing no less than 95% humidity. For hCMEC/D3 cells, ECM containing 5% fetal bovine serum (Sigma, USA) was utilized. MCF-10A cells were grown in DMEM (Sigma, USA), whereas the remaining cell lines were maintained in DMEM (Gibco, USA) supplemented with 10% fetal bovine serum (HyClone, USA). Regular testing confirmed that all the cell lines remained free from mycoplasma contamination.

### Cellular internalization of exosomes

Exosomes were labeled with DiIC16 (Gibco, USA) and subsequently resuspended in serum-free medium (FBS-SFM) supplemented with 10% exosome-depleted fetal bovine serum. This mixture was then added to immortalized hCMEC/D3 cells that had reached 80% confluence. Following an incubation period of 4 to 6 hours, imaging observations were conducted using a fluorescence microscope.

### Cell transfection

The designed short hairpin RNAs (shRNAs), small interfering RNAs (siRNAs), and inhibitors were produced by IGEBIO (Guangzhou, China) and subsequently transfected into the cells for a duration of 24 hours utilizing Lipofectamine 2000 (Invitrogen, Shanghai, China) per the supplier’s protocols. The RNA oligoribonucleotides are depicted in Supplementary Table 2.

### In vitro endothelial permeability assay

After constructing and treating a monolayer of vascular endothelial cells in a Transwell chamber, we quantified the amount of rhodamine-dextran labeled with FITC (MW 70,000; Sigma, USA) that passed through the endothelial cell monolayer to evaluate in vitro endothelial cell permeability.

### Western blot (wb) analysis

Total protein was extracted with RIPA lysis buffer (Sigma, USA), and the protein concentration was determined with a BCA protein detection kit (Elabscience, China). The extracted proteins were separated by sodium dodecyl sulfate‒polyacrylamide gel electrophoresis (SDS‒PAGE) and subsequently transferred onto polyvinylidene fluoride membranes (Millipore, USA). The membranes were incubated with antibodies against the following: tumor susceptibility gene 101 (TSG101, ab125011, Abcam, USA), cluster of differentiation 9 (CD9, ab236630, Abcam, USA), calnexin (ab22595, Abcam, USA), tight junction protein 1 (ZO-1, ab190085, Abcam, USA), occludin (ab216327, Abcam, USA), glucose transporter 1 (GLUT1, ab115730, Abcam, USA), 3-phosphoinositide-dependent protein kinase 1 (PDK1, ab202468, Abcam, USA), and phosphofructokinase-1, liver type (PFKL, ab241093, Abcam, USA). Afterward, the membrane was incubated with goat anti-rabbit H&L (HRP) (ab6721, Abcam, USA). Protein signals were visualized using a chemiluminescence system (Bio-Rad, SUA).

### Normal pcr and qPCR assays

Normal PCR and qPCR were conducted according to established protocols [28]. The primer sequences are listed in Supplementary Table 2. A LightCycler 480 from Roche was utilized for data collection and analysis.

### Apoptosis assay

An annexin V-FITC apoptosis detection kit (Beyotime, China) was used in this study. Cells (6 × 10^4^) were collected and suspended in 200 microliters of annexin V-FITC binding buffer. Subsequently, 5 microliters of the annexin V-FITC reaction solution and 5 microliters of propidium iodide (PI) were introduced at ambient temperature under dark conditions. After a 20-minute incubation phase at room temperature in the dark, the apoptotic cells were analyzed by flow cytometry (Beckman Coulter, USA).

### Migration and invasion assays

Established protocols were used to evaluate the invasive and migratory potential of BC cells [28]. For quantification, the cell counts were averaged across all five fields of view observed under the microscope.

### Ethynyl-2’-deoxyuridine (EdU) assay

After cells were plated on glass slides, 10 µmol/L 5-ethynyl-2’-deoxyuridine (EdU) was added. The assessment was performed utilizing the E-Click EdU Cell Proliferation Imaging Assay Kit (Elabscience, China). In accordance with the supplier’s protocol, the specimens were labeled with Alexa Fluor 488 and DAPI and then visualized under a fluorescence microscope (Olympus IX83, Japan). The percentage of proliferating cells was determined by computing the ratio of the number of EdU-positive nuclei to the overall number of nuclei.

### Immunofluorescence (if)

Cells for immunofluorescence were tested as previously described [28]. The cells were fixed with 4% paraformaldehyde and subsequently blocked by the addition of fetal bovine serum (FBS) in a dropwise manner. Afterward, the cells were incubated with antibodies targeting ZO-1 and occludin. The cells were then treated with fluorescent secondary antibodies (ab150080 ab150077, Abcam, USA), and DAPI (Sigma, USA) was added to stain the cell nuclei. Finally, images were acquired using a fluorescence microscope (Olympus IX83, Japan).

### Endothelial tube formation assay

Matrigel (Corning, USA) was diluted 1:1 and subsequently added to a 24-well plate. After being incubated at 37 °C for 1 to 2 hours, 75,000 treated immortalized human brain microvascular endothelial cells were introduced into each well. Following a culture period of 4 to 6 hours, the angiogenesis of the endothelial cells was observed under a microscope.

### In vitro BBB transmigration

A widely used in vitro blood-brain barrier (BBB) model was established using a co-culture system of hCMEC/D3 endothelial cells and the human glioblastoma line U87, which promotes a physiologically relevant BBB phenotype through U87-secreted factors [29–31]. For this model, 20,000 immortalized hCMEC/D3 cells were grown to confluence on Matrigel-coated inserts (8-μm pore; Corning, USA), with 5 × 10^4^ U87 cells cultured on the basolateral side. Twenty-four hours after reaching confluence, 5 × 10^5^ cancer cells were inoculated into the upper chamber of the insert. Over a period of 48 hours, the total number of DiIC16-labeled cancer cells that traversed both the endothelial cell layer and the human glioblastoma cell layer was counted in 10 fields of view. The DiIC16-stained migrated cells were subsequently fixed using a 10% formalin solution (Leica MZ10F, Germany).

### Dual-luciferase reporter assay

The wild-type sequence of the 3’ untranslated region (UTR) of the leukemia inhibitory factor receptor (LIFR), which contains the miR-221-3p binding site, and the mutant sequence of the 3‘UTR of LIFR, which harbors a mutation in the miR-221-3p binding site, were cloned and inserted into the pmirGLO vector to construct recombinant reporter plasmids. The wild-type LIFR 3‘UTR is designated the WT-LIFR 3‘UTR, while the mutant LIFR 3‘UTR is labeled the MUT-LIFR 3‘UTR. These reporter plasmids were transfected into hCMEC/D3 cells along with miR-221-3p or a negative control (miR-NC). Following a 48-hour incubation period, luciferase activity was measured using a dual-luciferase reporter gene detection system (Promega, USA). The pmirGLO vector was constructed by Vector Builder (Vector Builder Inc., Guangzhou). The 3‘UTRs and primers used for 3‘UTR cloning are listed in Supplementary Table 2.

### Glycolysis metabolism assay

In accordance with the supplier’s protocols, ATP production was measured using an ATP detection kit (Elabscience, China), glucose uptake was assessed with a glucose uptake detection kit (Elabscience, China), and lactate production was quantified using a lactate detection kit (Elabscience, China). These three parameters were utilized to evaluate the glycolytic metabolism of the cells.

### RNA-seq and Gene Set Enrichment Analysis

After vascular endothelial cells were treated with exosomes derived from various tumor cells, transcriptome sequencing and enrichment analysis were conducted on the cells using methods outlined in previously published articles [28].

### Metabolomic analyses

Metabolomic analysis was conducted by Suzhou PANOMIX Biomedical Co., Ltd. (Suzhou, China).

### Statistical analysis

Statistical analysis was conducted using SPSS version 20.0. Independent-samples t tests were employed to calculate *p* values for the in vitro experiments. To assess the correlation between variables, Spearman’s rank correlation analysis was performed. In all statistical analyses, two-sided *p* values were used. The experiments were independently repeated three times, yielding comparable results. Relevant representative data are presented in the figures and tables. Survival analysis utilizing Kaplan–Meier (KM) curves and log-rank statistical tests was performed to assess overall survival (OS) and survival outcomes of BCBM patients and their correlation with miR-221-3p expression levels.

## Results

### BC-Derived Exosomal miR-221-3p is associated with BCBM and Poor prognosis

**Fig. 1:**
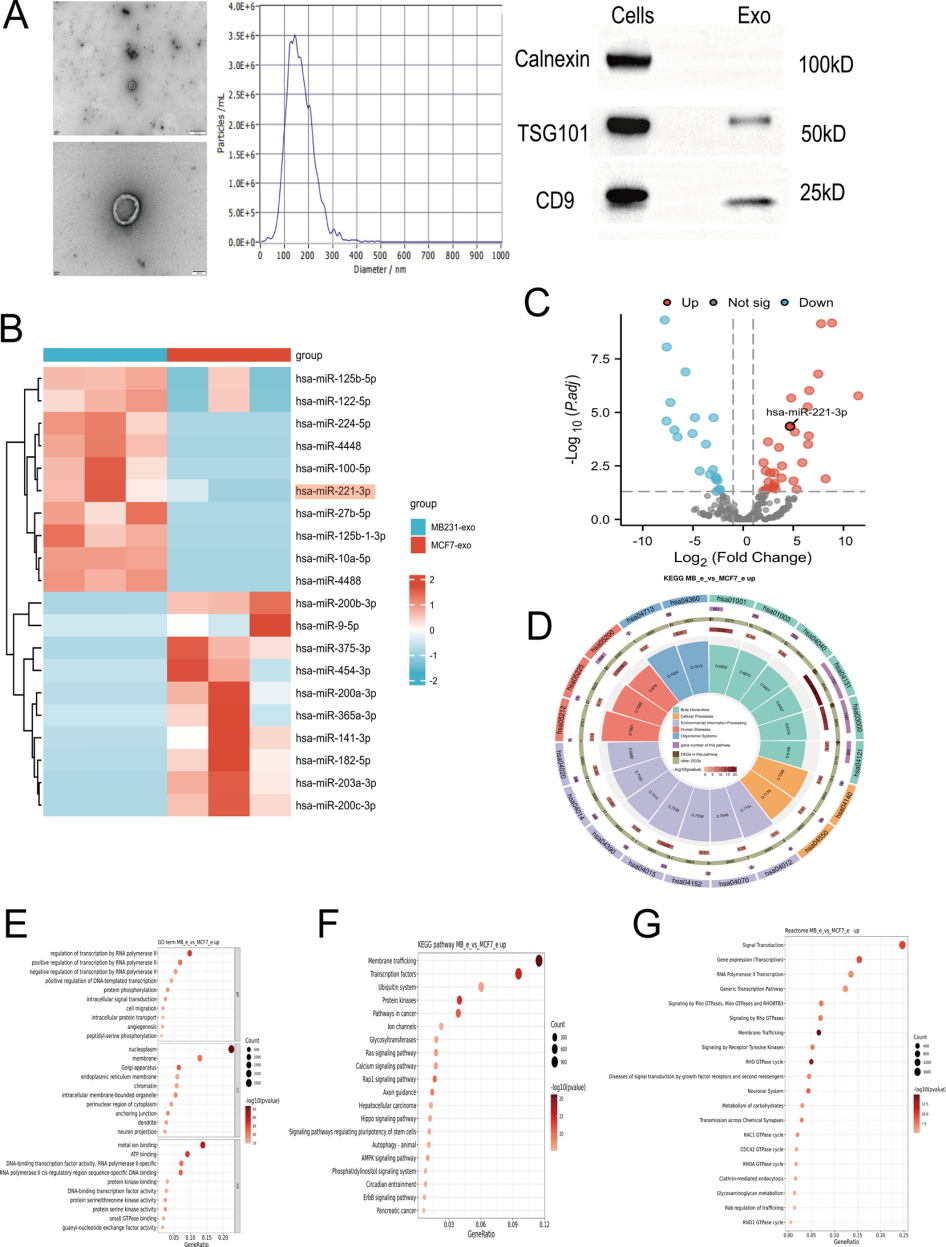
Isolation and characterization of breast cancer cell-derived exosomes. **A**) Transmission electron microscopy (tem), nanoparticle tracking analysis (nta), and Western blot analyses of exosomes derived from MDA-MB-231 cells. Scale bar: 500/100 nm. **B**) Heatmap representing the results of rna sequencing analyses of MB231-exos and MCF7-exos. **C**) Volcano plots illustrating the differentially expressed miRnas between MB231-exos and MCF7-exos. **D**) Loop of kegg enrichment analysis.**E-G**) Bubble plots depicting gene ontology (go), kegg, and reactome enrichment analyses of different miRnas between MB231-exos and MCF7-exos

To investigate the link between exosomal miRNAs and BCBM, we selected exosomes from the MDA-MB-231 (MB231-exos), MCF-7 (MCF7-exos), and MCF10A (MCF10A-exos) cell lines for our experiments. Exosomes were purified from conditioned medium using ultracentrifugation. Electron microscopy revealed that the exosomes exhibited a typical cup-shaped morphology, with sizes ranging from 30 to 150 nanometers. WB analysis was utilized to identify exosomal marker proteins. The results indicated that CD9 and tumor susceptibility gene 101 (TSG101) were abundant in the exosomes isolated from the cell supernatant, whereas calnexin, an exclusion marker for extracellular vesicles, was not detected (Fig. 1A).

We collected exosomes from metastatic MDA-MB-231 BC cells and from MCF-7 BC cells, which exhibit low metastatic potential, for miRNA sequencing analysis. The results indicated that compared with those in MCF7-exos, 220 types of miRNAs were increased and 130 types were decreased in MB231-exos. We focused on miR-221-3p, which exhibited relatively high expression (Fig. 1B–C). To explore more deeply the link between exosomal miRNAs and BCBM, we conducted Gene Ontology (GO), Kyoto Encyclopedia of Genes and Genomes (KEGG), and Reactome enrichment analyses of the sequencing results. We found that the top 20 enriched pathways were intimately linked to membrane transport and the negative modulation of transcription (Fig. 1D–G). Based on these experimental outcomes, we hypothesized that exosomal miR-221-3p derived from BC cells acts on brain-related cells to promote the occurrence of tumor brain metastasis.

**Fig. 2:**
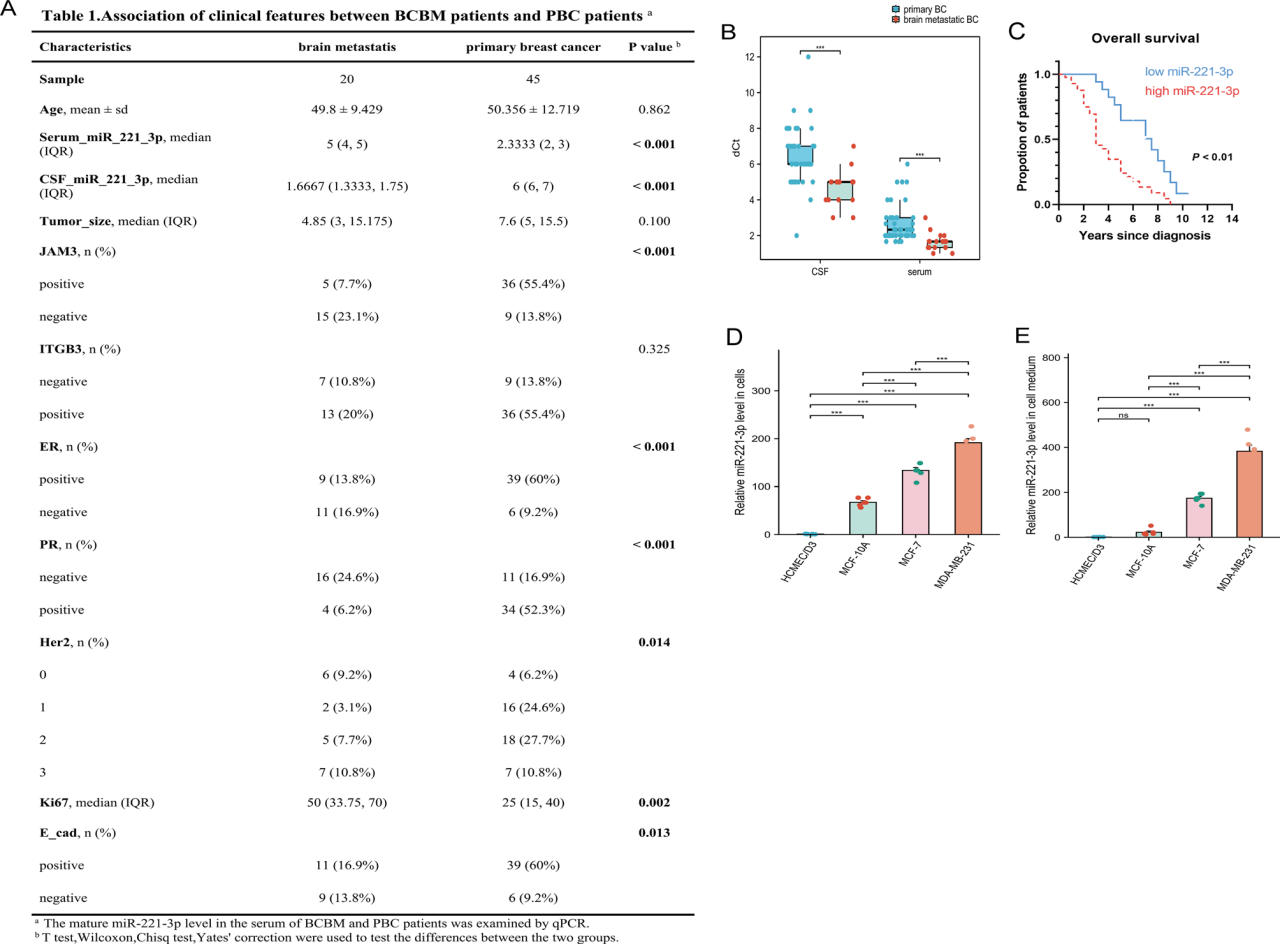
Breast Cancer Exosomal miR-221-3p Level is Associated with Breast Cancer, Brain Metastasis and Poor Prognosis. **A**) Association of clinical features between BCBM patients and PBC patients. **B**) The results of the qPCR experiment indicated that the levels of miR-221-3p in the serum and cerebrospinal fluid of BCBM patients were greater than those in the serum and cerebrospinal fluid of PBC patients. **c**) The serum level of miR-221-3p was negatively correlated with survival time. **D-E**) Metastatic breast cancer cells expressed and secreted high levels of miR-221-3p. The levels of miR-221-3p in hCMEC/D3, MDA-MB-231, MCF-7, and MCF-10A cells as well as in their corresponding conditioned media (cm) were analyzed by qPCR. ***, *p* < 0.001; ns, not significant

To assess the link between miR-221-3p and the occurrence of BCBM, we conducted qRT‒PCR analysis on serum and cerebrospinal fluid samples from 20 BC patients with brain metastasis (BCBM) and 45 BC patients with primary breast cancer (PBC) without metastasis, all of which were collected from the clinic (Fig. 2A). The results suggested that the serum and cerebrospinal fluid levels of miR-221-3p in the brain metastasis group were greater than those in the control group (Fig. 2B). Additionally, survival analysis revealed that individuals with BC with high serum levels of miR-221-3p had a significantly shorter survival time than those in the control group (Fig. 2C). Furthermore, we collected BC cells with varying metastatic potential and their conditioned medium (CM) for qRT‒PCR analysis. The findings suggested that the miR-221-3p level in both tumor cells and CM was positively correlated with the metastatic potential of the tumor cells (Fig. 2D–E).

### BC-Derived exosomal miR-221-3p can Be taken up by hCMEC/D3 cells

**Fig. 3:**
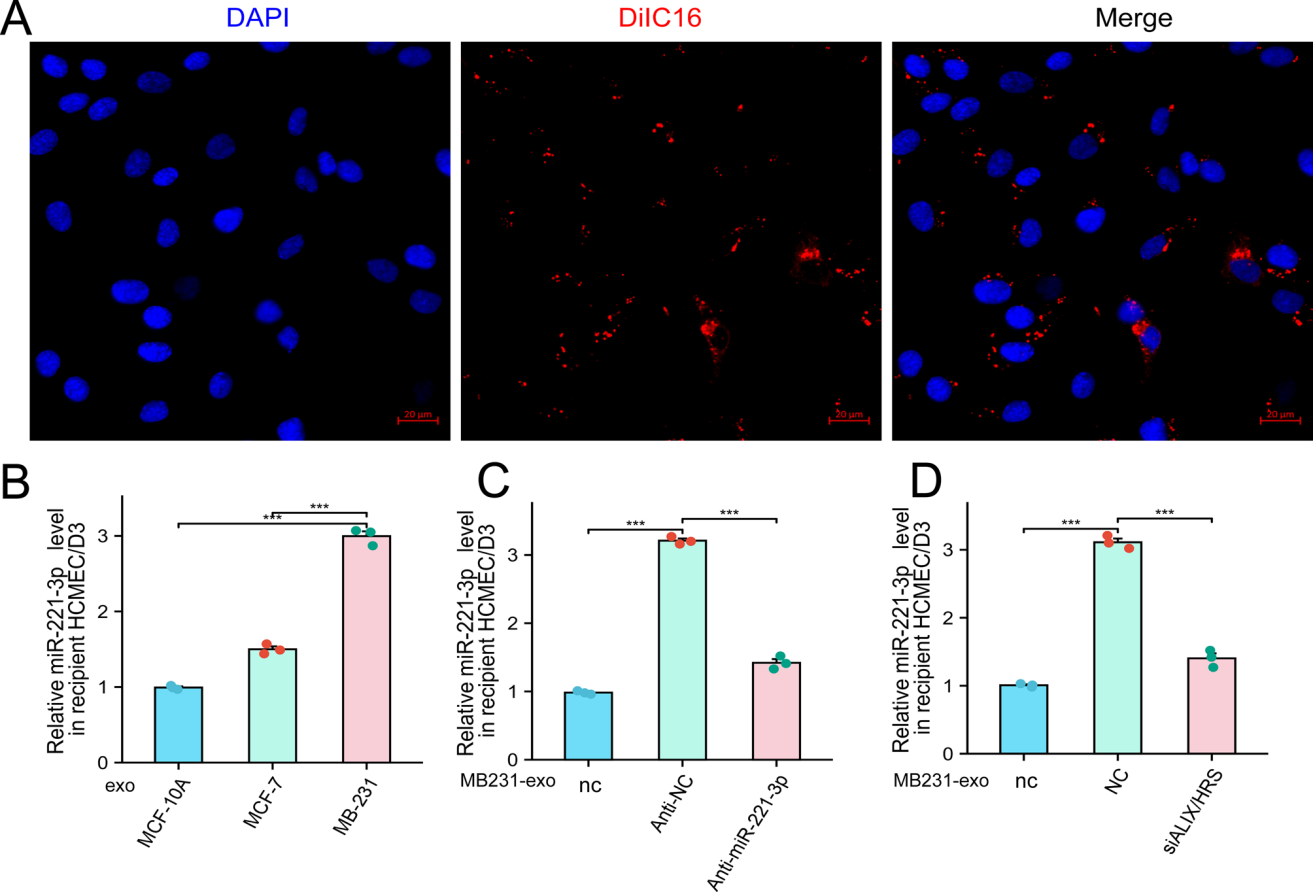
Breast cancer cell-derived miR-221-3p is delivered into hCMEC/D3 cells via exosomes. **A**) DiIC16-labeled exosomes derived from MDA-MB-231 cells were internalized by hCMEC/D3 cells. Scale bars represent 20 μm. **B**) The level of miR-221-3p significantly increased in recipient hCMEC/D3 cells incubated with MB231-exos. HCMEC/D3 cells were cultured with exosomes derived from MDA-MB-231, MCF-7, or MCF-10A cells. **c**) Antagonizing endogenous miR-221-3p in MDA-MB-231 cells diminished the ability of MDA-MB-231-derived exosomes to increase miR-221-3p levels in recipient hCMEC/D3 cells. hCMEC/D3 cells were cultured with or without exosomes derived from either anti-NC- or anti-miR-221-3p-transfected MDA-MB-231 cells. **D**) Inhibition of exosome secretion from MDA-MB-231 cells reduced the ability of MDA-MB-231-derived exosomes to increase miR-221-3p levels in recipient hCMEC/D3 cells. hCMEC/D3 cells were cultured with or without exosomes from either nc or siALIX/HRS-transfected MDA-MB-231 cells, with 25 nM of each siRNA being transfected. **For B-D**) hCMEC/D3 cells were cultured without (-) or with the indicated exosomes for 24 hours before being subjected to qPCR analysis to assess miR-221-3p levels. ***, *p* < 0.001; nc, negative control

Given that tumor cell migration across the BBB represents a crucial and fundamental step in brain metastasis progression, our investigation focused on brain microvascular endothelial cells. We began by coculturing DiIC16-labeled MB231-exos with immortalized human brain microvascular endothelial cells (hCMEC/D3 cells) and noted that MB231-exos were internalized by the hCMEC/D3 cells (Fig. 3A). Next, we exposed hCMEC/D3 cells to MB231-exos, MCF7-exos, and MCF10A-exos independently. qRT‒PCR revealed that miR-221-3p expression in hCMEC/D3 cells was directly correlated with miR-221-3p expression in the exosome-producing cells (Fig. 3B). When hCMEC/D3 cells were treated with exosomes obtained from anti-miR-221-3p-transfected MDA-MB-231 BC cells, our data revealed no substantial increase in the hCMEC/D3 cell miR-221-3p level compared with that in the control group (anti-NC) (Fig. 3C). These observations suggest tumor cells as the probable source of miR-221-3p in the hCMEC/D3 cells. To investigate the exosome-mediated interaction between BC cells and hCMEC/D3 cells more thoroughly, we suppressed ALIX and HRS expression in MDA-MB-231 cells to restrict tumor exosome production. After obtaining conditioned media and extracting exosomes using identical procedures, we exposed hCMEC/D3 cells and discovered that the miR-221-3p level remained relatively unchanged compared with that in the control group (Fig. 3D). These findings establish that hCMEC/D3 cells can increase miR-221-3p levels through the internalization of BC cell-derived exosomes.

### BC-Derived exosomal miR-221-3p promotes glycolysis in hCMEC/D3 cells

**Fig. 4:**
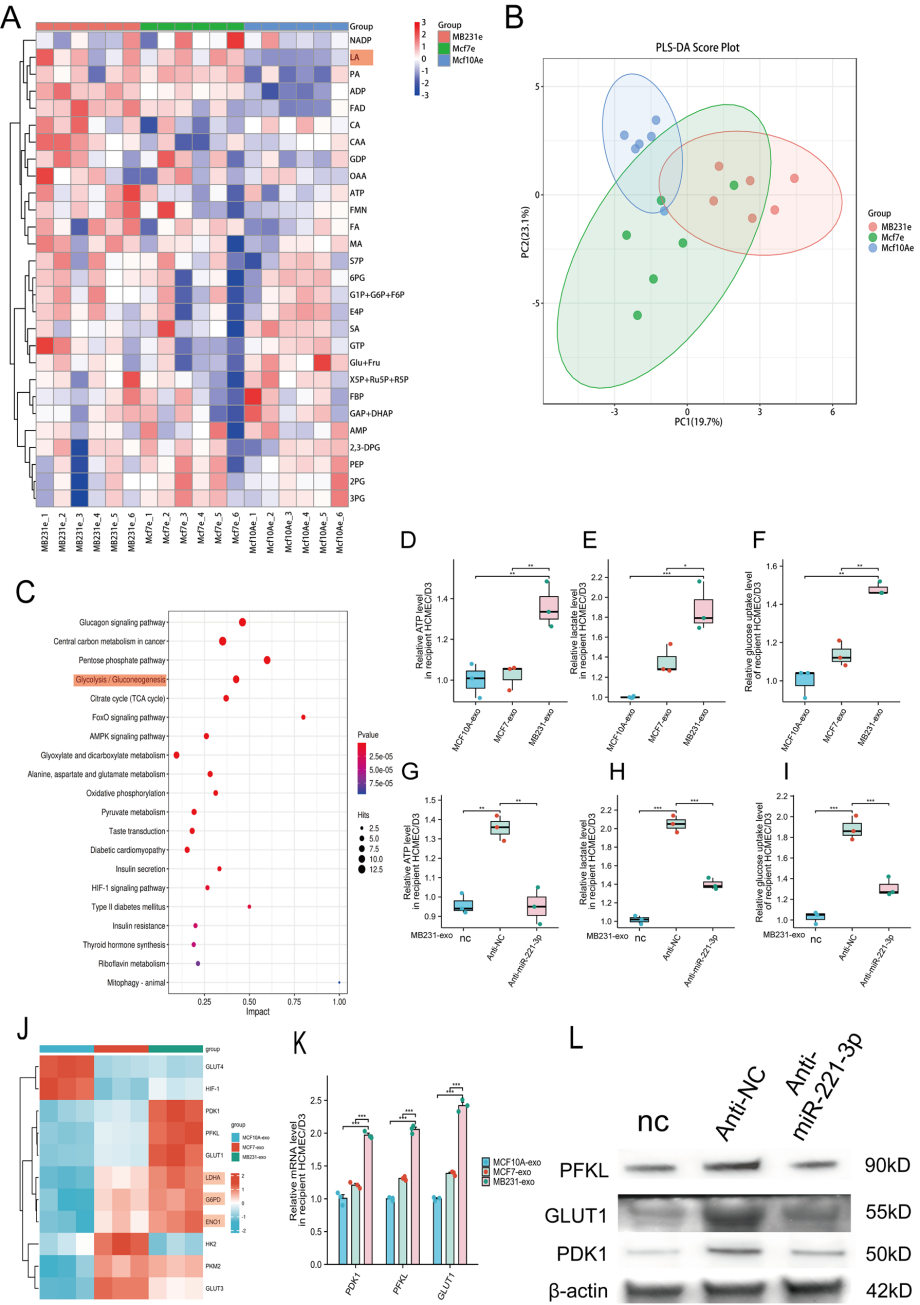
Breast cancer-derived micells were internalizedR-221-3p promotes glycolysis in hCMEC/D3 cells. **A**) Heatmap of the results of the metabolomic analyses of hCMEC/D3 cells. hCMEC/D3 cells were cultured with exosomes derived from MDA-MB-231, MCF-7, and MCF-10A cells for 48 hours. **B**) PLS‒DA score plot of hCMEC/D3 cells cultured with exosomes derived from MDA-MB-231, MCF-7, and MCF-10A cells. **C**) Bubble plots illustrating the results of the kegg enrichment analyses of the metabolomic data. **D-F**) The levels of atp production, glucose uptake, and lactate production were significantly increased in hCMEC/D3 cells cultured with exosomes from MDA-MB-231 cells. **G-I**) The levels of atp production, glucose uptake, and lactate production were significantly decreased in hCMEC/D3 cells following miR-221-3p inhibition. **J**) Heatmap of the results of rna sequencing analyses focusing on key glycolytic enzymes in hCMEC/D3 cells cultured with different exosomes. **K**) Validation of three key enzymes exhibiting the most significant differences, as assessed by qRT–PCR in hCMEC/D3 cells cultured with various types of exosomes. **l**) Western blot analysis revealed that the expression of three key enzymes was significantly reduced in hCMEC/D3 cells after miR-221-3p inhibition. Anti-NC- or anti-miR-221-3p-transfected hCMEC/D3 cells were cultured with or without MB231-exos for 48 hours prior to Western blot analysis. *, *p* < 0.05; **, *p* < 0.01; ***, *p* < 0.001; nc, negative control

To further examine the impact of BC-derived exosomal miR-221-3p on the metabolic functions of hCMEC/D3 cells, we treated hCMEC/D3 cells with MB231-exos, MCF7-exos, or MCF10A-exos and subsequently conducted a metabolomic analysis. The results indicated that the levels of glycolytic metabolites were markedly increased in the MB231-exo- and MCF7-exo-treated groups, particularly in the MB231-exo-treated group, compared with those in the MCF10A-exo-treated group (Fig. 4A). Furthermore, the PLS‒DA score plot suggested notable intergroup differences in the metabolites among the three cell groups (Fig. 4B). We also performed KEGG enrichment analysis of the metabolomic data and found that among the top 20 enriched pathways, several were closely related to the cellular glycolysis process (Fig. 4C).

To further investigate the effect of BC-derived exosomal miR-221-3p on the glycolytic process in hCMEC/D3 cells, we used kits to measure glucose uptake, lactate production, and ATP production levels in hCMEC/D3 cells treated with three different types of exosomes. The results indicated that compared with those in the other two groups, the glucose uptake, lactate production, and ATP production levels in the MB231-exo-treated group markedly elevated (Fig. 4D–F). Furthermore, transfection of anti-miR-221-3p into hCMEC/D3 cells treated with MB231-exos resulted in notable decreases in glucose uptake, lactate production, and ATP production levels compared with those in the control group (Fig. 4G–I), corroborating the findings from our metabolomic analysis.

To further investigate the mechanism by which BC-derived exosomal miR-221-3p regulates the glycolytic process in hCMEC/D3 cells, we conducted a transcriptomic sequencing analysis of hCMEC/D3 cells treated with three types of exosomes. We assessed the expression patterns of essential enzymes that participate in glycolysis. Among the ten key glycolytic enzymes, PDK1, PFKL, and GLUT1 exhibited markedly increased expression in hCMEC/D3 cells treated with MB231-exos (Fig. 4J). We subsequently performed qRT‒PCR and WB analyses to confirm these observations (Fig. 4K–L). The validation outcomes were aligned with the transcriptomic findings. These results indicate that BC-derived exosomal miR-221-3p may increase the cellular glycolytic rate by indirectly modulating the expression of key glycolytic enzymes in hCMEC/D3 cells.

### BC-Derived exosomal miR-221-3p promotes the proliferation, migration and angiogenic ability of hCMEC/D3 cells while inhibiting apoptosis

**Fig. 5:**
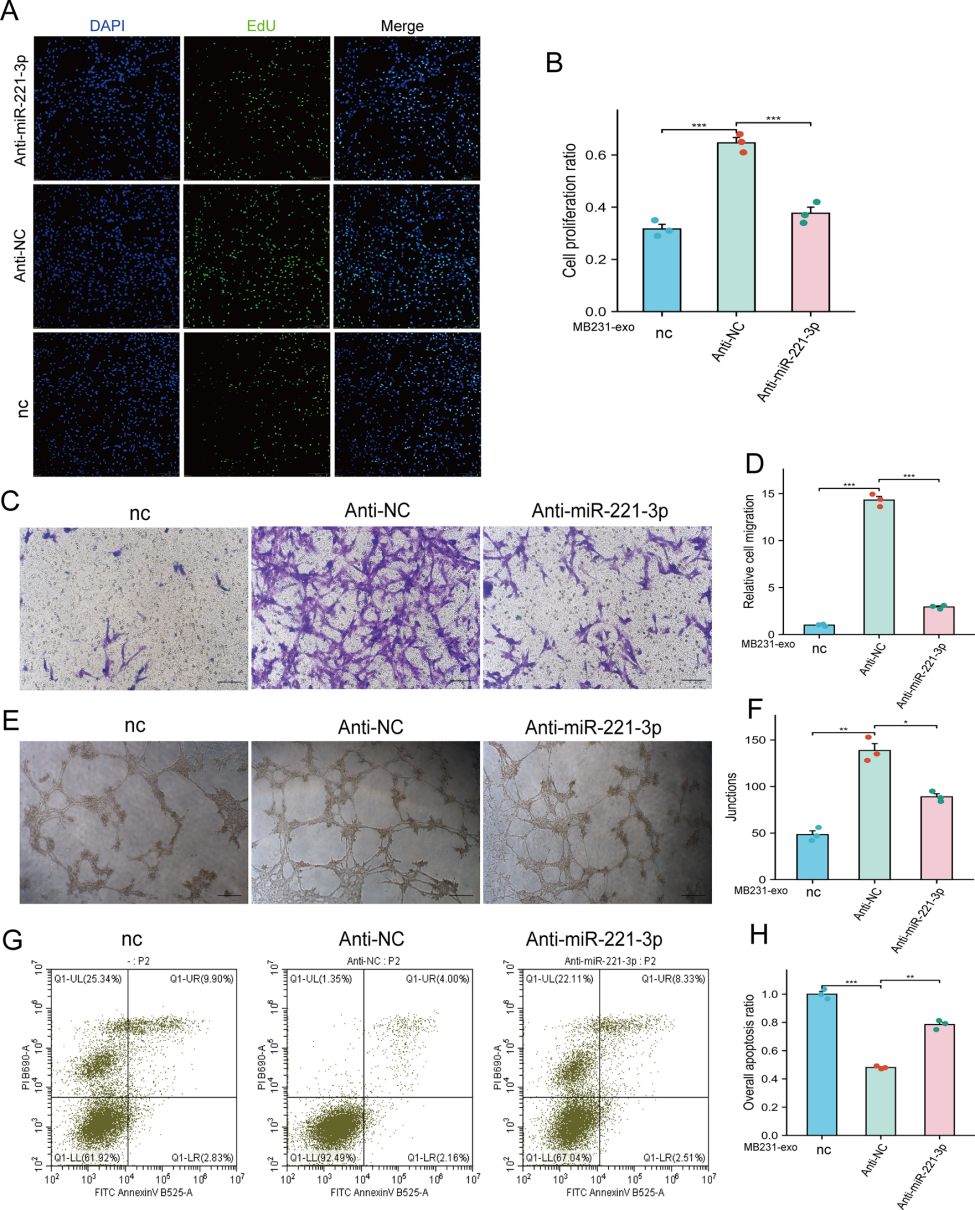
Breast cancer-derived miR-221-3p promotes the proliferation, migration and angiogenic ability of hCMEC/D3 cells while inhibiting apoptosis. **A-B**) An EdU assay revealed that antagonizing miR-221-3p in hCMEC/D3 cells attenuated the ability of MB231-exos to increase cell viability. Scale bars represent 200 μm. **C - D**) The antagonistic effect of miR-221-3p on hCMEC/D3 cells attenuated their migratory ability. Scale bars represent 75 μm. **E - F**) the antagonism of miR-221-3p in hCMEC/D3 cells weakened the junctions of hCMEC/D3 cells. Scale bars represent 75 μm. **G - H**) The antagonistic effect of miR-221-3p on hCMEC/D3 cells led to an increase in the overall apoptosis ratio of hCMEC/D3 cells. For (**A-H**), anti-NC- or anti-miR-221-3p-transfected hCMEC/D3 cells were cultured with or without MB231-exos for 48 hours. *, *p* < 0.05; **, *p* < 0.01; ***, *p* < 0.001; nc, negative control

To further investigate whether the alterations in glycolysis in hCMEC/D3 cells induced by BC-derived exosomal miR-221-3p result in functional changes in hCMEC/D3 cells that facilitate tumor metastasis, we transfected anti-miR-221-3p into hCMEC/D3 cells treated with MB231-exos and subsequently performed cell proliferation, Transwell migration, and angiogenesis assays. The results demonstrated that the inhibition of miR-221-3p in hCMEC/D3 cells markedly counteracted the effects of MB231-exos on promoting proliferation (Fig. 5A–B), migration (Fig. 5C–D), and angiogenesis (Fig. 5E–F) in hCMEC/D3 cells. In the apoptosis assay, the antagonistic effect of miR-221-3p on hCMEC/D3 cells effectively diminished the ability of MB231-exos to suppress apoptosis in hCMEC/D3 cells (Fig. 5G–H). The observed changes in the proliferation, migration, angiogenesis, and apoptosis of hCMEC/D3 cells may contribute to the remodeling of the BBB.

### BC-Derived exosomal miR-221-3p remodels the permeability of the in vitro BBB and Promotes transendothelial tumor cell migration

**Fig. 6:**
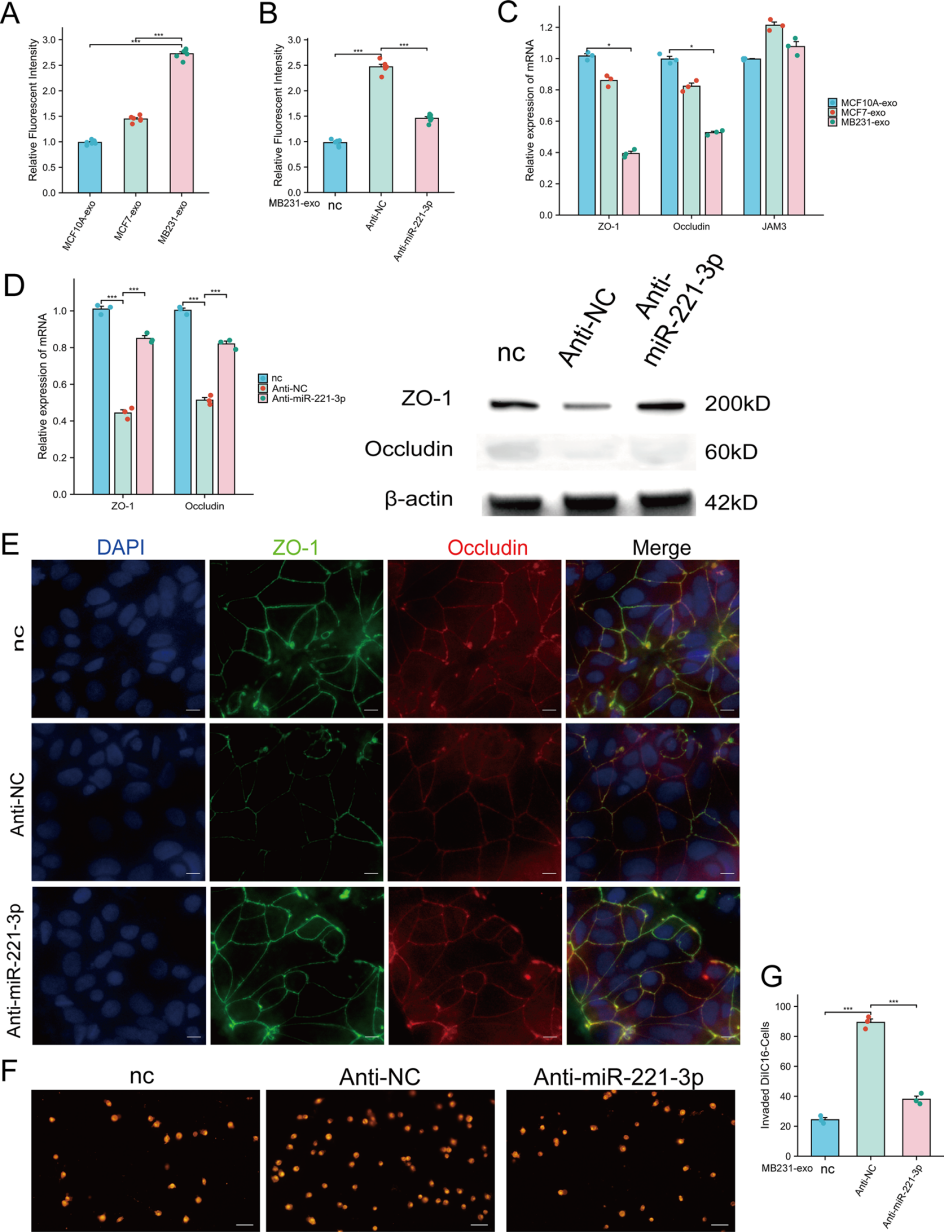
Breast cancer-derived miR-221-3p increases the permeability of endothelial monolayers and facilitates the transendothelial invasion of tumor cells. **A**) hCMEC/D3 cell monolayers pretreated with MB231-exos exhibited increased permeability. **B**) Antagonism of miR-221-3p in hCMEC/D3 cells reduced the ability of MB231-exos to enhance endothelial permeability. hCMEC/D3 cells were transfected with either anti-NC or anti-miR-221-3p, subsequently reseeded on transwell inserts, and incubated with or without MB231-exos. **C**) qRT‒PCR revealed that the expression levels of ZO-1 and occludin were downregulated in hCMEC/D3 cells treated with MB231-exos. **D**) Antagonism of miR-221-3p in hCMEC/D3 cells diminished the ability of MB231-exos to reduce the expression of ZO-1 and occludin. **E**) Immunofluorescence staining revealed that the antagonistic effect of miR-221-3p on hCMEC/D3 cells diminished the ability of MB231-exos to decrease ZO-1 and occludin expression. Scale bar, 25 μm. **F-G**) Antagonizing miR-221-3p in hCMEC/D3 cells reduced the ability of MB231-exos to facilitate the transendothelial invasion of DiIC16-labeled MDA-MB-231 cells. Scale bar, 75 μm. For (**B, D-F**), anti-NC- or anti-miR-221-3p-transfected hCMEC/D3 cells were cultured with or without MB231-exos for 48 hours. *, *p* < 0.05; **, ***, *p* < 0.001; nc, negative control

Increased permeability of the BBB is a contributing factor to the occurrence of brain metastases in tumors. To further examine whether BC-derived exosomal miR-221-3p can induce changes in the permeability of an in vitro BBB model, we constructed this model using Matrigel and hCMEC/D3 cells in a Transwell chamber. The resulting in vitro BBB models were treated with three types of exosomes, and equal amounts of rhodamine-dextran were subsequently added to the chamber. Changes in permeability were assessed by measuring the amount of rhodamine-dextran that permeated through the in vitro BBB. The results indicated that the permeability of the in vitro BBB model was markedly increased by treatment with MB231-exos (Fig. 6A). Furthermore, knocking down miR-221-3p in hCMEC/D3 cells effectively reversed the ability of MB231-exos to enhance the permeability of the in vitro BBB (Fig. 6B).

Tight junction proteins between vascular endothelial cells are closely linked to the permeability of the BBB. In this study, we evaluated ZO-1, occludin, and JAM3 levels in hCMEC/D3 cells treated with exosomes using qRT‒PCR analysis. The results indicated a decrease in the expression of ZO-1 and occludin after treatment with MB231-exos, whereas JAM3 expression remained unchanged (Fig. 6C). Additionally, we found that knocking down the level of miR-221-3p in hCMEC/D3 cells effectively inhibited the ability of MB231-exos to reduce the expression levels of ZO-1 and occludin in these cells. Consistent results were also observed in WB and immunofluorescence experiments (Fig. 6D–E).

To investigate whether the alteration in the permeability of the BBB model induced by BC-derived exosomal miR-221-3p contributes to an increase in tumor cell transmigration across the BBB, we introduced DiIC16-labeled MDA-MB-231 BC cells into the treated BBB model. The results indicated that the knockdown of miR-221-3p in hCMEC/D3 cells markedly decreased the number of BC cells that traversed the in vitro BBB model (Fig. 6F–G). These observations demonstrate that BC-derived exosomal miR-221-3p could accelerate the development of BC metastasis by increasing BBB permeability.

### Lifr is a functional target of miR-221-3p

**Fig. 7:**
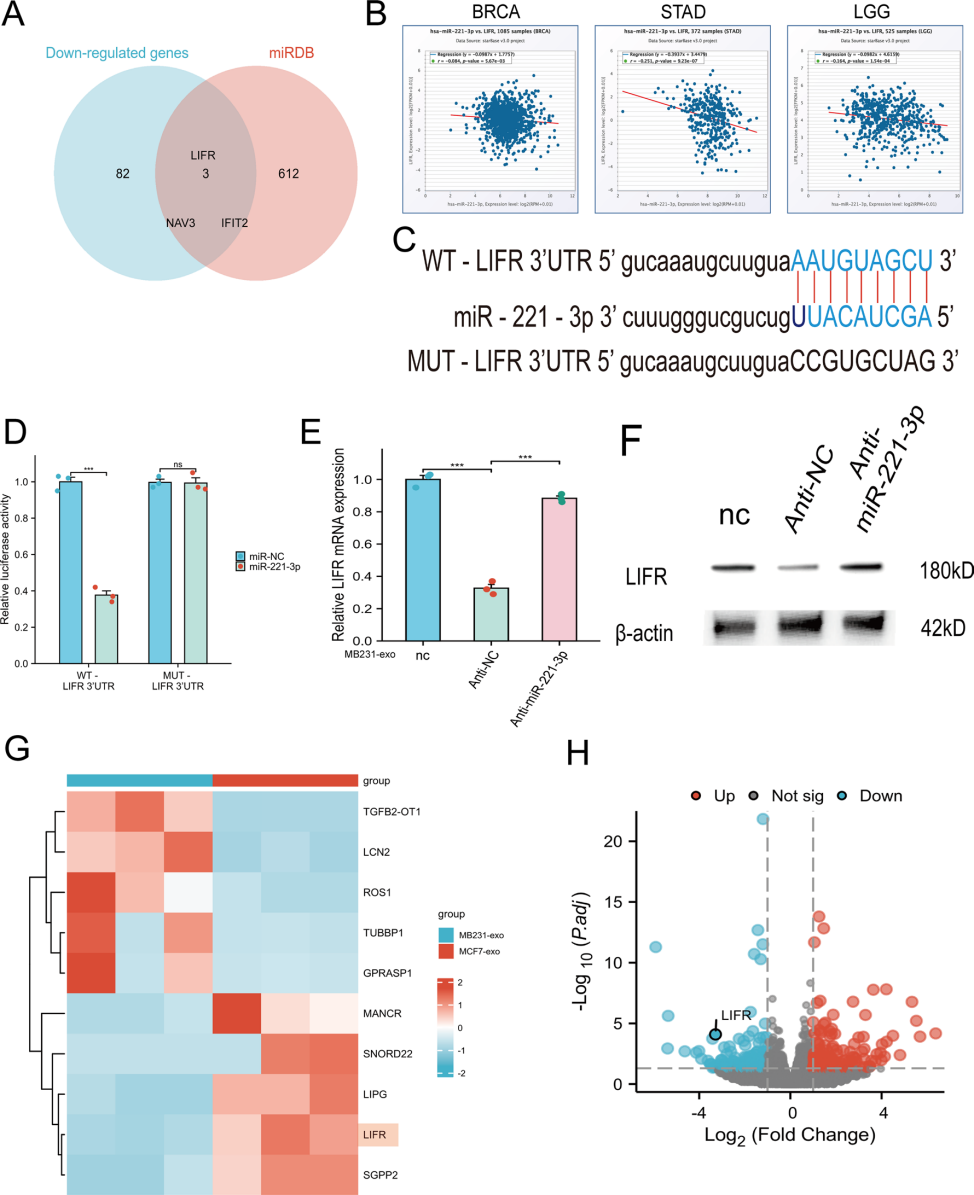
Lifr is a common target of miR-221-3p. **A**) a Venn diagram was constructed to identify mRNAs targeted by miR-221-3p. **B**) Pancancer analysis of miR-221-3p and lifr was conducted across three types of cancers. **C**) The binding sites between the lifr 3’UTR and miR-221-3p are depicted. For (**B-C**), The data were sourced from the starBase database. **D**) luciferase activity was significantly lower in hCMEC/D3 cells cotransfected with miR-221-3p and the WT-LIFR 3ʹUTR than in those cotransfected with the MUT-LIFR 3ʹUTR. **E-F**) Antagonism of miR-221-3p in hCMEC/D3 cells diminished the ability of MB231-exos to reduce lifr expression levels, as assessed by qRT‒PCR and Western blot analysis. **G**) A heatmap of the results of rna sequencing analyses of hCMEC/D3 cells cultured with various types of exosomes is presented. **H**) Differentially expressed genes are illustrated using volcano plots. ***, *p* < 0.001; nc, negative control; ns, not significant

To identify the potential mRNAs targeted by miR-221-3p, we screened 615 potential targets using the miRBD database. Concurrently, transcriptomic analysis revealed that 85 mRNAs were expressed at lower levels in hCMEC/D3 cells treated with MB231-exos than in those treated with MCF7-exos. By intersecting these two datasets, we identified only three common mRNAs: LIFR, NAV3, and IFIT2 (Fig. 7A). We subsequently focused on LIFR.

To examine the connection between miR-221-3p and LIFR, we utilized the starBase database and identified an inverse correlation between the expression of miR-221-3p and LIFR in breast invasive carcinoma (BRCA), stomach adenocarcinoma (STAD), and low-grade glioma (LGG) (Fig. 7B). Additionally, we predicted the binding sites of miR-221-3p on LIFR mRNA using the starBase database (Fig. 7C). Subsequently, a dual-luciferase reporter gene assay revealed that coexpressing miR-221-3p substantially reduced the activity of the firefly luciferase reporter gene containing the wild-type 3‘UTR of LIFR. Importantly, this suppressive outcome was eliminated when mutations were introduced to the predicted binding regions in the 3‘UTR (Fig. 7D).

After the knockdown of miR-221-3p in hCMEC/D3 cells, we performed qRT‒PCR analysis and WB experiments. The results indicated that the knockdown of miR-221-3p effectively inhibited the capacity of MB231-exos to decrease the expression of LIFR in hCMEC/D3 cells (Fig. 7E–F). These findings align with the results obtained from our RNA sequencing analyses (Fig. 7G–H). Collectively, these results suggest that LIFR may be a direct functional target of miR-221-3p.

### Knocking down lifr again in hCMEC/D3 cells with antagonized miR-221-3p can restore the functional impact of miR-221-3p on hCMEC/D3 cells

**Fig. 8:**
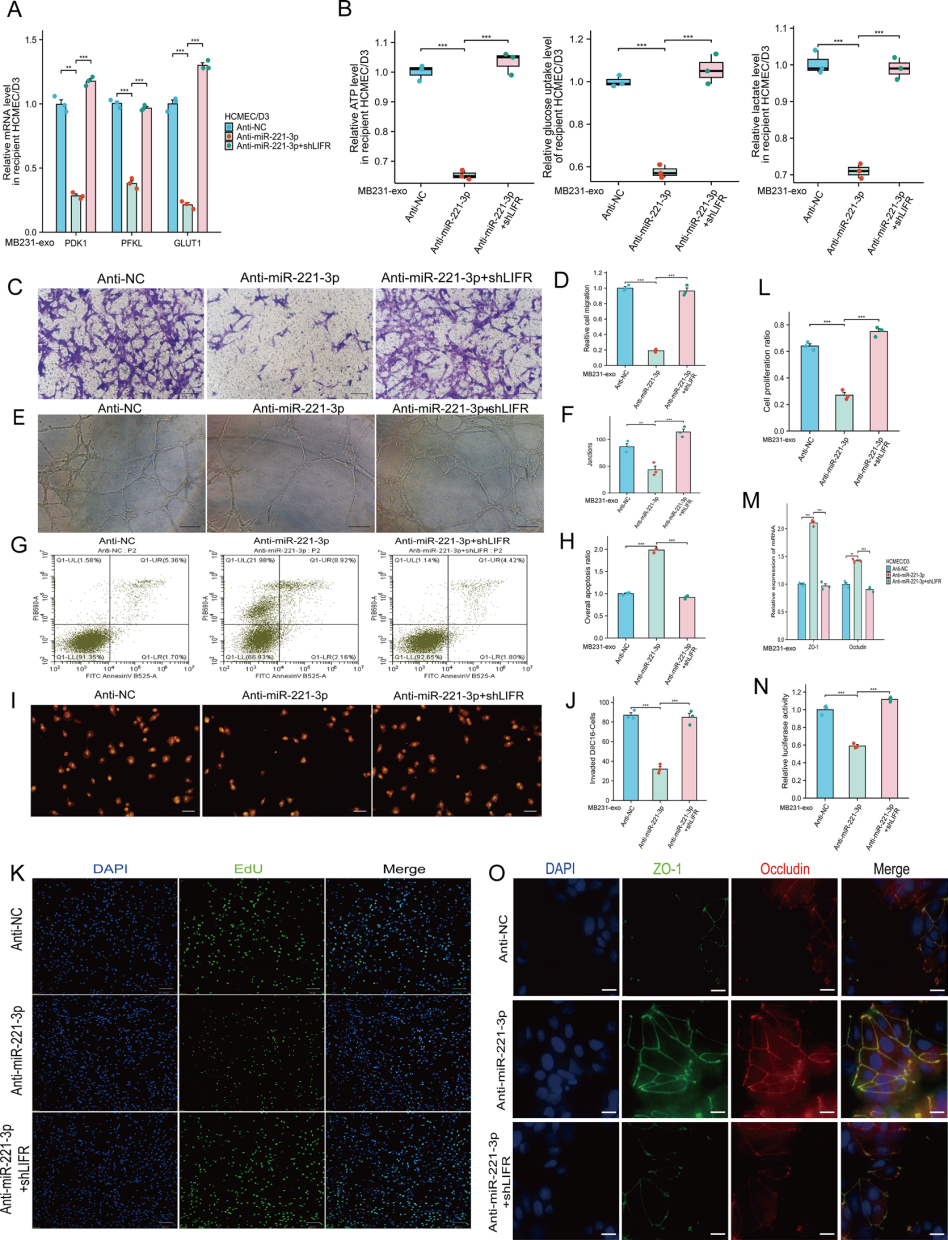
Downregulation of lifr expression reverses the effects of anti-miR-221-3p in hCMEC/D3 cells. **A**) The antagonistic effect of miR-221-3p on hCMEC/D3 cells reduced the ability of MB231-exos to increase the expression of key glycolytic enzymes. However, cotransfection with shLIFR and anti-miR-221-3p reversed these effects. **B**) The transfection of shLIFR counteracted the effect of anti-miR-221-3p in hCMEC/D3 cells, leading to increases in atp production, glucose uptake, and lactate production. **C-D**) shLIFR transfection in hCMEC/D3 cells reversed the ability of anti-miR-221-3p to suppress the migratory capacity of these cells. **E-F**) in vitro tube formation assays revealed that shLIFR transfection in hCMEC/D3 cells restored the ability of anti-miR-221-3p to disrupt the junctions formed by hCMEC/D3 cells. **G-H**) The antagonistic effect of miR-221-3p on hCMEC/D3 cells increased the overall apoptosis rate, but cotransfection with shLIFR and anti-miR-221-3p reversed this effect. **I-J**) The antagonism of miR-221-3p in hCMEC/D3 cells diminished the ability of MB231-exos to promote the transendothelial invasion of DiIC16-labeled MDA-MB-231 cells, an effect that was reversed by cotransfection with shLIFR and anti-miR-221-3p. **K-L**) The results of the EdU assay demonstrated that antagonizing miR-221-3p expression in hCMEC/D3 cells reduced the ability of MB231-exos to increase cell viability, and cotransfection of shLIFR and anti-miR-221-3p reversed this effect. Scale bars represent 200 μm. m) Anti-miR-221-3p counteracted the MB231-exo-mediated reduction in ZO-1 and occludin expression, and concurrent shLIFR transfection reversed this effect. **N**) Antagonizing miR-221-3p in hCMEC/D3 cells decreased the ability of MB231-exos to increase endothelial permeability, an effect that was also reversed by shLIFR transfection. **O**) Immunofluorescence staining indicated that antagonizing miR-221-3p in hCMEC/D3 cells led to increases in ZO-1 and occludin expression levels, which were reversed by the cotransfection of shLIFR and anti-miR-221-3p. Scale bar, 25 μm. **, *p* < 0.01; ***, *p* < 0.001; ns, not significant

To further investigate the relationships among BC-derived exosomal miR-221-3p, LIFR, and the functions of hCMEC/D3 cells, we cotransfected anti-miR-221-3p and shLIFR into hCMEC/D3 cells, and the changes in the protein expression of LIFR in hCMEC/D3 cells were confirmed by WB analysis (Supplementary Figure 1C). Compared with that in cells transfected with anti-miR-221-3p alone, the expression of key glycolytic enzymes in the cotransfected cells was markedly restored (Fig. 8A, Supplementary Fig. 1D). Additionally, the levels of ATP production, glucose uptake, and lactate production were all markedly increased (Fig. 8B).

The results of the proliferation, migration, angiogenesis, and apoptosis experiments demonstrated that the transfection of hCMEC/D3 cells with anti-miR-221-3p alone markedly inhibited the ability of MB231-exos to promote migration (Fig. 8C–D), angiogenesis (Fig. 8E–F), and proliferation (Fig. 8K–L) in but also inhibited apoptosis (Fig. 8G–H). However, LIFR knockdown in hCMEC/D3 cells with suppressed miR-221-3p expression effectively reversed these effects.

Next, we conducted qRT‒PCR analysis on hCMEC/D3 cells in three groups: the control group transfected with anti-NC; the group transfected solely with anti-miR-221-3p; and the group cotransfected with anti-miR-221-3p and shLIFR. The results indicated that the cotransfection of anti-miR-221-3p and shLIFR markedly restored the ability of MB231-exos to reduce the expression levels of ZO-1 and occludin in hCMEC/D3 cells (Fig. 8M, Supplementary Fig. 1E). Similar findings were also observed by immunofluorescence (Fig. 8O).

Finally, we reconstructed the in vitro BBB to conduct permeability experiments and assess tumor transmigration across this barrier. The results indicated that the ability of MB231-exos to increase the permeability of the in vitro BBB and facilitate the transmigration of BC cells was markedly restored in the cotransfection group (Fig. 8I–J, N). These findings suggest that BC-derived exosomal miR-221-3p may directly target the LIFR protein, collectively promoting the occurrence of tumor brain metastasis by influencing the glycolysis of hCMEC/D3 cells and the expression of tight junction proteins.

## Discussion

Exosomes facilitate interactions between BC cells and cellular components of the brain metastasis microenvironment, thereby promoting tumor metastasis. In our study, we demonstrated that brain metastasis in BC can be enhanced by BC cells secreting exosomal miR-221-3p, which acts on hCMEC/D3 cells. Mechanistically, we revealed a dual regulatory axis through which exosomal miR-221-3p functions simultaneously. Specifically, miR-221-3p induces the upregulation of glycolytic activity in hCMEC/D3 cells by modulating the translation of LIFR. Additionally, miR-221-3p influences the expression of tight junction proteins by targeting LIFR, which increases the permeability of the BBB. These two mechanisms collectively promote the occurrence of BCBM. To the best of our knowledge, this is the first study to investigate the role of BC cell-derived exosomes in inducing glycolysis in brain microvascular endothelial cells to facilitate cancer progression. Previous research has also indicated that BC-derived exosomal miR-221-3p contributes to chemoresistance in tumors, corroborating our findings that the expression of miR-221-3p in BC cell-derived exosomes is elevated [32, 33]. Our study further elucidates the role of miR-221-3p upregulation in BC cell-derived exosomes and clarifies the underlying mechanisms, providing new insights into the interaction between BC cell-derived exosomes and cellular components in the brain metastasis microenvironment while highlighting the significant role of BC-derived exosomal miR-221-3p in promoting cancer progression.

The role of BC cell-derived exosomes in the progression of BC has been extensively studied. Research has confirmed that these exosomes can facilitate metastasis to the liver [34, 35], lungs [36, 37], bones [38, 39], and brain [40, 41]. For instance, Weiying Zhou et al. demonstrated that BC-derived exosomal miR-105 directly interferes with the expression of the tight junction protein ZO-1 in vascular endothelial cells, promoting tumor metastasis to the lungs and brain [10]. Similarly, Xinxin Yuan et al. reported that BC-derived exosomal miR-21 can induce the differentiation and proliferation of osteoclasts within the premetastatic niche, thereby enhancing the occurrence of tumor bone metastasis [42]. Additionally, significant progress has been made in understanding how exosomes contribute to BC chemoresistance [43, 44] and immune evasion [35, 45]. For example, Mingli Han et al. confirmed that exosomal AFAP1–AS1 from trastuzumab-resistant BC cells can induce drug resistance in nonresistant cells [46]. Furthermore, Samantha M. Morrissey et al. reported that BC cell-derived exosomes can induce macrophages in the premetastatic niche to polarize toward an immunosuppressive phenotype, thereby promoting tumor metastasis [47]. In this study, we found that BC-derived exosomal miR-221-3p regulates glycolysis and the expression of tight junction proteins in hCMEC/D3 cells, promoting tumor metastasis. Antagonizing miR-221-3p in tumor and endothelial cells or inhibiting exosome generation markedly weakens this effect, indicating that exosomal miR-221-3p may be a key factor in the occurrence of BCBM. The significance of exosomal miR-221-3p in regulating vascular endothelial cells during cancer progression also warrants further investigation in other cancer types.

To date, several studies have demonstrated that BC cell-derived exosomes are capable of modulating glycolysis in target cells. For instance, Miranda Y. Fong and colleagues reported that miR-122 within BC cell-derived exosomes can influence the glycolytic activity of cells in the metastatic microenvironment, thereby facilitating tumor metastasis [24]. Similarly, Samantha M. Morrissey et al. reported that BC cell-derived exosomes can affect the glycolysis of macrophages in the metastatic microenvironment, leading to the polarization of macrophages into an immunosuppressive phenotype and promoting tumor metastasis [47]. In our research, we revealed that BC-derived exosomal miR-221-3p can target LIFR in hCMEC/D3 cells, resulting in increased glycolytic capacity and reduced expression of tight junction proteins. These combined effects contribute to the occurrence of tumor metastasis. Previous investigations have also suggested that the regulatory role of LIFR in glycolysis is markedly important in the progression of gastric cancer [48], pancreatic cancer, and lung cancer [49], which corroborates our findings.

Recent studies have confirmed that the upregulation of glycolytic activity in endothelial cells can influence the expression of tight junction proteins between cells [50, 51]. For instance, Zhengjun Hou et al. demonstrated that lactic acid can activate the TGF-β pathway, leading to the induction of endothelial‒mesenchymal transition (EndoMT) and resulting in the downregulation of tight junction protein expression [52]. However, research in this area concerning brain microvascular endothelial cells remains limited. In our study, we observed that the TGF-β pathway was activated in the hCMEC/D3 cells in the MB231-exo treatment group compared with the MCF7-exo treatment group (Supplementary Figure 1A-B). Furthermore, extensive research has shown that activation of the TGF-β pathway can promote the occurrence of EndoMT [53, 54]. These findings suggest that the decreased expression of tight junction proteins in hCMEC/D3 cells may be associated with enhanced glycolysis and the occurrence of EndoMT. This represents an intriguing and promising research direction that warrants further in-depth investigation.

The potential universality of the miR-221-3p/LIFR pathway is underscored by pan-cancer evidence showing consistent miR-221-3p upregulation and inversely correlated LIFR expression across malignancies (Fig. 7B), with functional roles established in hepatocellular and gastric carcinomas [48, 55]. Our study extends this paradigm by demonstrating that breast cancer-derived exosomal miR-221-3p remotely disrupts the brain vascular niche via LIFR-mediated glycolytic activation and tight junction impairment. The clinical relevance of this mechanism is supported by our finding of significantly elevated miR-221-3p levels in serum and cerebrospinal fluid from patients with brain metastases compared to those with primary breast cancer (Fig. 2B). However, the activity of this axis may vary across molecular subtypes of breast cancer, reflecting underlying tumor heterogeneity. Our current clinical analysis, while confirming the association with metastasis, did not assess its variation across molecular subtypes (e.g., Luminal A/B, HER2+, TNBC). Similarly, our cellular models, which established the core mechanism across lines with varying metastatic potential, may not capture the full heterogeneity of miR-221-3p expression and function across the spectrum of triple-negative subtypes. Therefore, elucidating the personalized dimensions of this regulatory axis—through expanded subtype-stratified cohorts and diverse cellular models—represents a critical direction for future research.

Another key consideration for interpreting these mechanisms is the cellular composition of our in vitro BBB model, which employed a co-culture of immortalized hCMEC/D3 endothelial cells and U87 glioblastoma cells but did not include pericytes [29–31]. While existing evidence suggests that incorporating pericytes may not confer significant functional benefits in such models [56, 57], and their absence does not affect our core conclusions, we acknowledge this as a simplification of the in vivo complexity. Future studies utilizing tri-culture systems that include pericytes will help validate these findings under more physiologically relevant conditions.

## Conclusions

In conclusion, our findings indicate that BC can target LIFR in hCMEC/D3 cells via exosomal miR-221-3p, thereby promoting glycolysis and inhibiting the expression of tight junction proteins, which facilitates tumor metastasis. This research reveals a novel interaction between BC cell-derived exosomes and brain microvascular endothelial cells and highlights a new role for miR-221-3p in the progression of BC.

## Electronic supplementary material

Below is the link to the electronic supplementary material.

Supplementary Material 1

Supplementary Material 2

Supplementary Material 3

## Data Availability

The datasets used and/or analyzed during the current study are available from the lead contact on reasonable request.

## Abbreviations and nomenclature

BCBM: Breast cancer brain metastasis
BC: Breast cancer
LIFR: Leukemia inhibitory factor receptor
TNBC: Triple-negative breast cancer
miRNAs: MicroRNAs
hCMEC/D3: Immortalized brain microvascular endothelial cells
FBS-SFM: Serum-free medium
shRNAs: Short hairpin RNAs
siRNAs: Small interfering RNAs
TSG101: Tumor susceptibility gene 101
CD9: Cluster of differentiation 9
ZO-1: Tight junction protein 1
GLUT1: Glucose transporter 1
PDK1: 3-phosphoinositide-dependent protein kinase 1
PFKL: Phosphofructokinase-1, liver type
GO: Gene Ontology
KEGG: Kyoto Encyclopedia of Genes and Genomes
CM: Conditioned medium
BRCA: Breast invasive carcinoma
STAD: Stomach adenocarcinoma
LGG: Low-grade glioma
EndoMT: Endothelial‒mesenchymal transition
