# Supplementary material for: Tumor exosomal miR-221-3p induces glycolysis through the LIFR/GLUT1 pathway to destroy the cerebral vascular endothelial cell barrier and promote breast cancer brain metastasis
Source: J Transl Med. 2025 Nov 21;23:1333. doi: 10.1186/s12967-025-07372-8 (PMC12639775; doi:10.1186/s12967-025-07372-8)
Supplement: Supplementary file 1 — Supplementary Material 1 [file 12967_2025_7372_MOESM1_ESM.docx]

**Tumor exosomal miR-221-3p induces glycolysis through the LIFR/GLUT1 pathway to destroy the cerebral vascular endothelial cell barrier and promote breast cancer brain metastasis**

Kaitao Zhu^1,2^, Hongru Yao^1,2^, Jilong Hei^1,2^, Shiwei Li^3^,Tongxin Ye^1,2,^

WenG Jiang ^4^, Shuwen Wang^5^ , Zhuojun Luo^6^, Tracey Martin^4^, Jie Zhou^6🖂^, Shanyi Zhang^1,2,4🖂^

1. Department of Neurosurgery, Sun Yat-Sen Memorial Hospital, Sun Yat-Sen University, Guangzhou, China.
2. Guangdong Provincial Key Laboratory of Malignant Tumor Epigenetics and Gene Regulation, Guangdong-Hong Kong Joint Laboratory for RNA Medicine, Sun Yat-Sen Memorial Hospital, Sun Yat-Sen University, Guangzhou, China.
3. Department of Neurosurgery, HeYou International Health System, Foshan, China.
4. Cardiff China Medical Research Collaborative (CCMRC), (Cardiff University - Peking University Cancer Institute and Cardiff University - Capital Medical University Joint Centre Biomedical Research), Cardiff University, School of Medicine, Cardiff, United Kingdom.
5. Department of Neurosurgery, The People's Hospital of Fengqing, Lincang, China.
6. Department of Breast Oncology Surgery，Guangzhou Institute of Cancer Research, the Affiliated Cancer Hospital, Guangzhou Medical University，Guangzhou, 510095，China.

🖂：Corresponding author.

🖂Jie Zhou zhoujie833@gzhmu.edu.cn

🖂Shanyi Zhang [zsyscience@163.com](mailto:zsyscience@163.com)

**Inventory of supplementary data**

1. Supplementary Figure and legend………....…...….…..Page 3

2. Supplementary Table………………….……....…….…Page 4

3. Supplementary Materials and Methods……….……… Page 5-7


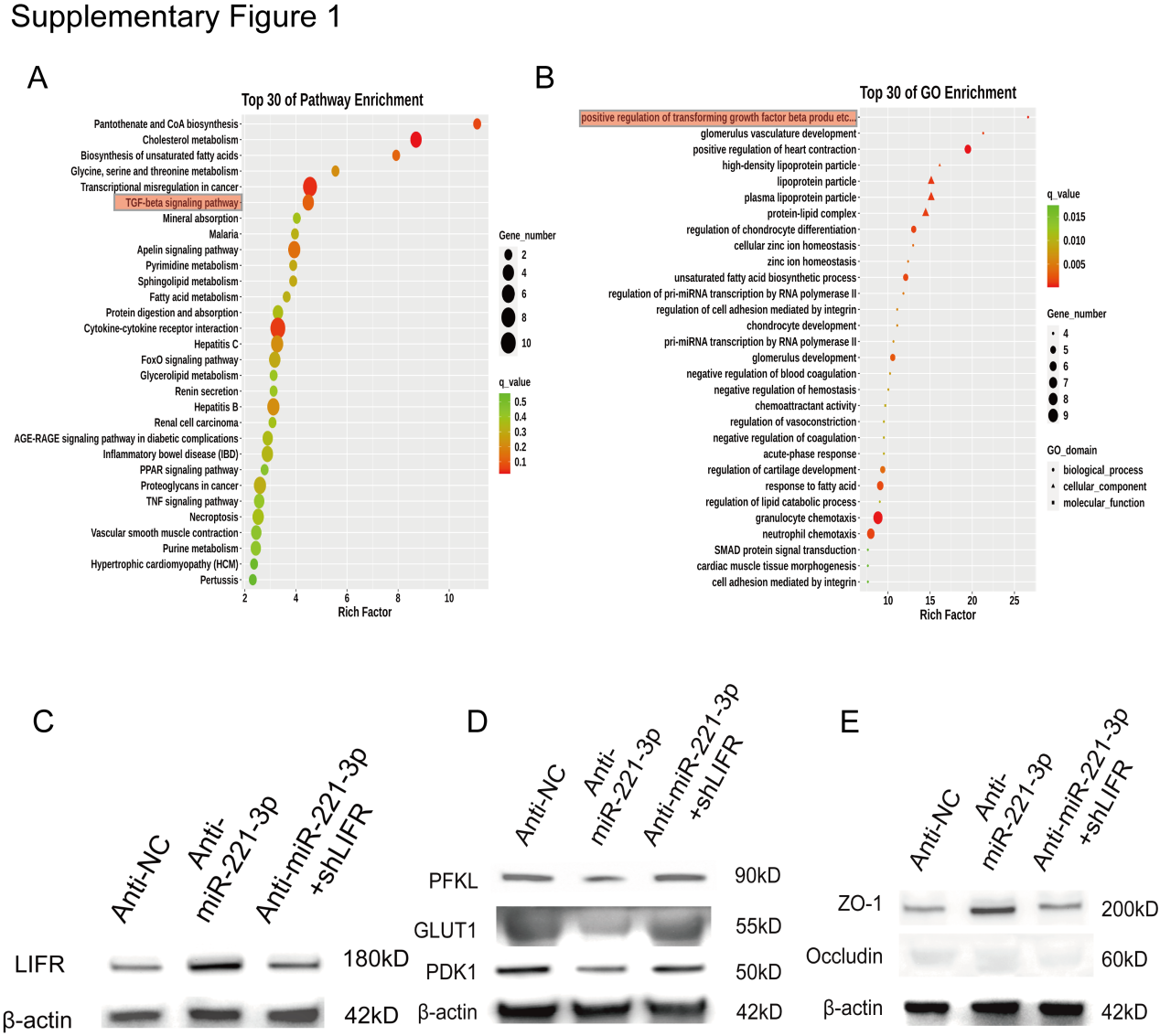


**Supplementary Figure 1. A-B)** Bubble plots depict Gene Ontology (GO) and KEGG enrichment analyses of different genes in hCMEC/D3, cultured with exosomes derived from MDA-MB-231 and MCF-7 cells for 48 hours. **C)** miR-221-3p inhibition attenuated the MB231-exo-induced downregulation of LIFR expression, an effect rescued by shLIFR co-transfection. **D)** miR-221-3p antagonism suppressed MB231-exo-driven increase of key glycolytic enzymes, which was similarly restored through shLIFR co-transfection. **E)** Anti-miR-221-3p counteracted MB231-exo-mediated reduction of ZO-1 and Occludin, with concurrent shLIFR transfection reversing this modulation. **For (C-E)** HCMEC/D3 cells were transfected with Anti-NC, Anti-miR-221-3p, or Anti-miR-221-3p combined with shLIFR for 24 hours, followed by incubation with exosomes derived from MB231-exo for 48hours.


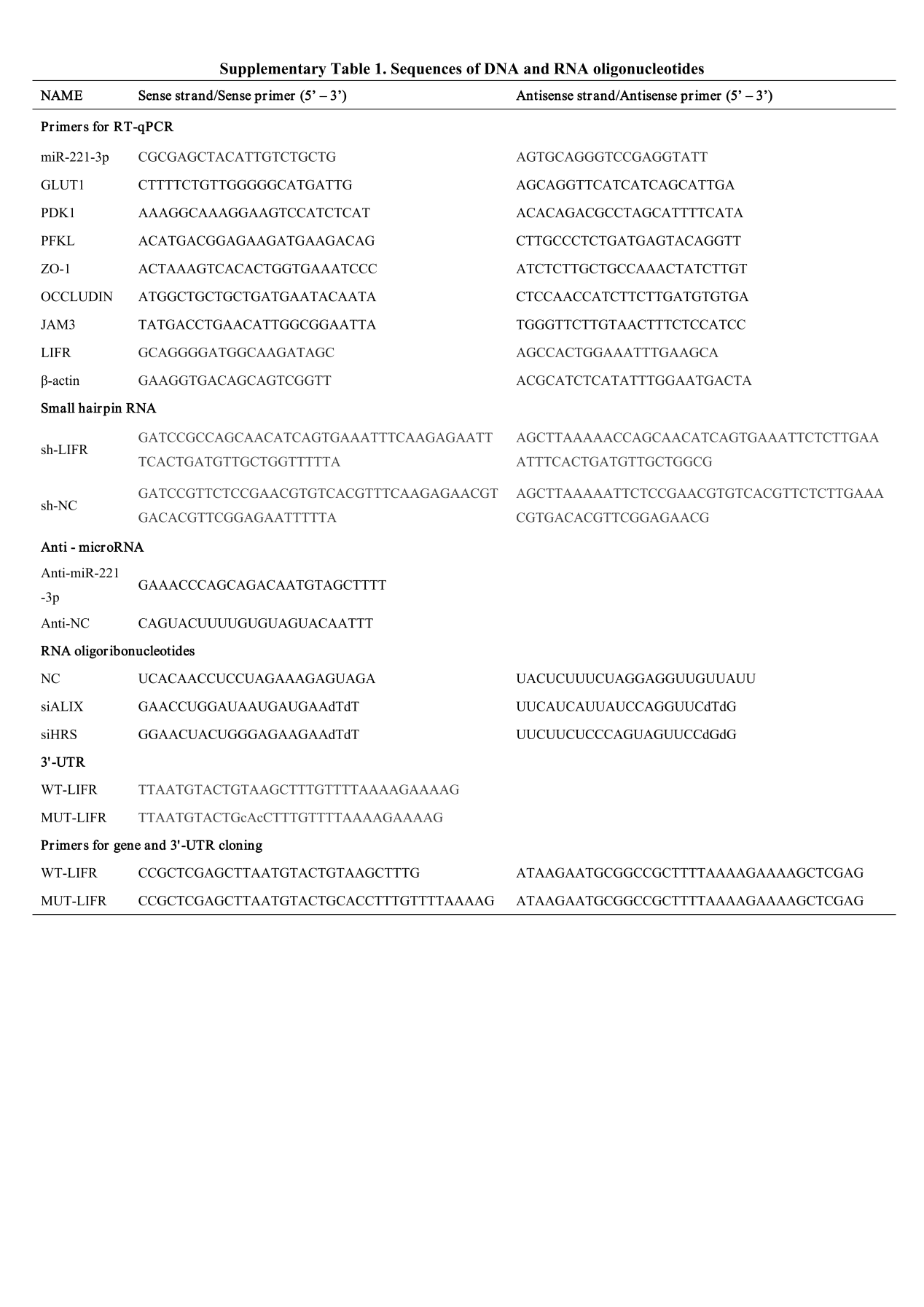


**Supplementary Materials and Methods**

### Patients and Ethics

All breast tumor and BCBM samples were procured from Sun Yat-sen Memorial Hospital of Sun Yat-sen University between March 2012 and December 2023. Immediately following surgery, the patient specimens were preserved in liquid nitrogen and subsequently transferred to a -80 °C freezer for storage until needed. And the serum and cerebrospinal fluid were similarly tested as previously described. The Research Ethics Committee of Sun Yat-sen Memorial Hospital of Sun Yat-sen University approved all protocols and procedures, and informed consent was acquired from the patients.

### EV Isolation

FBS-free conditioned medium (CM) was collected from cells cultured for 48 hours. Dead cells and contaminating debris were eliminated through centrifugation at 500 g for 15 minutes, succeeded by an additional centrifugation step at 12,000 g for 30 minutes at 4 °C. The supernatant was subsequently processed via ultracentrifugation at 120,000 g for 70 minutes at 4 °C. The resulting pellet underwent PBS washing, and another round of ultracentrifugation was performed under the same conditions to obtain a final pellet. This pellet was then resuspended in PBS and further purified using an exosome purification kit (Echobiotech, China). For cell treatment, we employed a BCA protein concentration detection kit (Elabscience, China) for protein quantification. Cell extracellular vesicles with a protein content of 2 micrograms, which is equivalent to those derived from approximately 5 × 10⁶ producing cells, were added to 2 × 10⁵ recipient cells for co-culture, with a duration of 24 hours for qPCR experiments and 48 hours for functional experiments.

### Nanoparticle Tracking Analysis

Nanoparticle tracking analysis (NTA) was executed to evaluate the dimensions and quantity of exosomes at VivaCell Shanghai, implementing the Zetaview-PMX120-Z (Particle Metrix, Meerbusch, Germany) alongside its ZetaView software (version 8.05.14 SP7). The obtained exosome specimens underwent suitable dilution with 1 × phosphate-buffered saline (Beyotime, China) before examination. NTA readings were documented and examined across 11 separate positions. The ZetaView instrument underwent calibration using 110-nanometer polystyrene particles. During the examination procedure, the temperature remained between 23 °C and 30 °C.

### Transmission Electron Microscopy

The exosome specimens were adjusted to an appropriate concentration. Subsequently, 15 µL of the specimen was deposited onto a copper grid and maintained for 2 minutes. Following this, approximately 15 µL of a 2% uranyl acetate staining solution at room temperature was added using a pipette and allowed to act for 1 minute. The samples were then dried under an incandescent lamp for 3 minutes. The structural features and dimensions of the exosomes were assessed and captured utilizing transmission electron microscopy (JEM1400, Japan).

### Metabolomics Analyses

Transfer a suitable quantity of the sample to a 2 mL centrifuge tube, then accurately add 500 μL of methanol and vortex to mix thoroughly. Place the tube in the adapter of the grinder and immerse it in liquid nitrogen for 5 minutes. Following this, perform a freeze-thaw cycle at room temperature and shake the sample at 55 Hz for 1 minute. Transfer the tube to a thermostatic metal shaker and shake at 1500 rpm at 4 °C for 10 minutes. After shaking, centrifuge the mixture at 12000 rpm at 4 °C for 10 minutes. Next, pipette 100 μL of the supernatant into another 2 mL centrifuge tube, add an appropriate amount of the mixed internal standard, and vortex thoroughly. Subsequently, dry the solution using nitrogen in a fume hood. Reconstitute the sample with a 50% acetonitrile/water solution, vortex thoroughly, and centrifuge again at 12000 rpm for 10 minutes at 4 °C. Finally, transfer the supernatant into an injection vial for analysis.
