# Supplementary figures and images for: Tumor exosomal miR-221-3p induces glycolysis through the LIFR/GLUT1 pathway to destroy the cerebral vascular endothelial cell barrier and promote breast cancer brain metastasis
Source: J Transl Med. 2025 Nov 21;23:1333. doi: 10.1186/s12967-025-07372-8 (PMC12639775; doi:10.1186/s12967-025-07372-8)

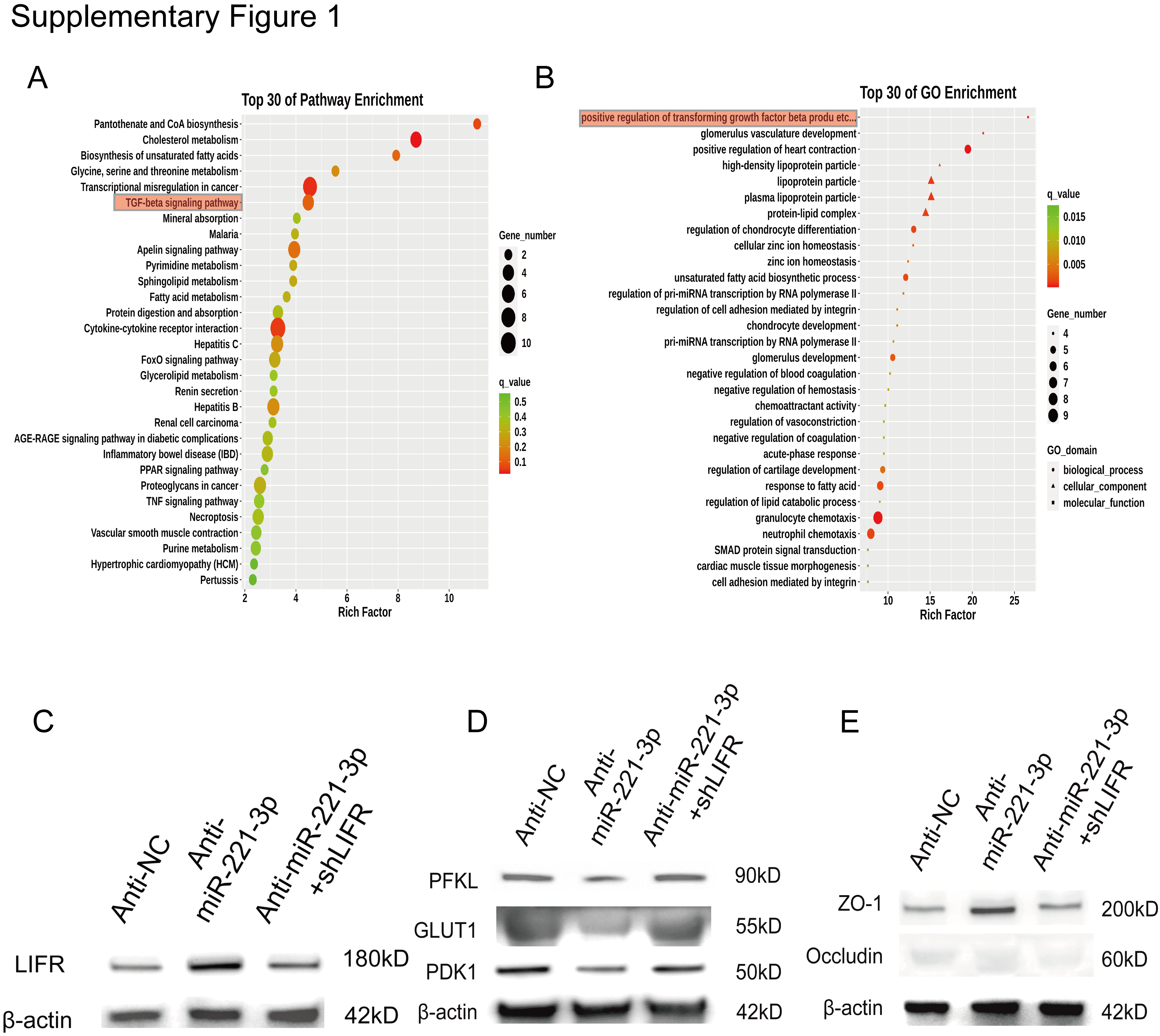

Supplement: Supplementary file 2 — Supplementary Material 2 [file 12967_2025_7372_MOESM2_ESM.tif]
